# Philadelphia Department of Health Doula Support Program: Early Successes and Challenges of a Program Serving Birthing People Affected by Substance Use Disorder

**DOI:** 10.1007/s10995-023-03803-4

**Published:** 2023-11-09

**Authors:** Nadia Haerizadeh-Yazdi, My-Phuong Huynh, Arielle Narva, Amirah Grasty, MaryNissi Lemon, Nick Claxton, Kelly Gillespie, Stacey Kallem

**Affiliations:** Philadelphia Department of Public Health, Division of Maternal, Child and Family Health, 1101 Market Street, Philadelphia, 19107 United States

**Keywords:** Doula care, Substance use disorder, Perinatal, Postpartum, Local health department

## Abstract

**Purpose:**

Maternal substance use and deaths due to overdoses are increasing nationwide. Evidence suggests that the rate of resumed substance use, and fatal and non-fatal overdose is greatest in the first year after birth, particularly around six months postpartum, compared to other parts of the perinatal period. Doula care has been linked to improvements in perinatal health and outcomes.

**Description:**

In response to the opioid epidemic, the Philadelphia Department of Public Health developed and implemented the Doula Support Program (DSP), with a focus on one year of postpartum care for birthing people with a substance use disorder (SUD). In this paper, we describe the program and its formation and report on the early challenges and successes of the program implementation, based on information we received from program founders and managers in a group interview.

**Assessment:**

Early successes of the program include partnering with local community-based programs to recruit and retain doulas, supplementing traditional doula education with perinatal SUD-specific trainings, and maximizing client referrals by collaborating with local organizations and treatment centers that serve birthing people with SUD. Client retention, however, has proven to be challenging, especially during the COVID-19 pandemic.

**Conclusion:**

The DSP continues to grow, and lessons learned will facilitate program improvements. The goal of this paper is to outline the development and launch of the DSP and to act as a model for other state and local health departments interested in providing doula care for birthing people with SUD.

**Supplementary Information:**

The online version contains supplementary material available at 10.1007/s10995-023-03803-4.

## Significance

Maternal substance use and fatal overdoses are increasing in Philadelphia, Pennsylvania. In response to this crisis, the Philadelphia Department of Public Health’s Division of Maternal, Child and Family Health developed the Doula Support Program (DSP), offering doula care to pregnant people with a current or past SUD history up to one-year postpartum. To our knowledge, this is the only doula program in Pennsylvania offering one-year postpartum support to birthing people affected by SUD.

**Supplementary Information:**

The online version contains supplementary material available at 10.1007/s10995-023-03803-4.

## Purpose

Philadelphia is one of several epicenters of the nation’s opioid epidemic and has one of the highest overdose mortality rates compared to other large cities (Health of the City Report, 2021). Between 2009 and 2016, 25% of pregnancy-associated deaths were due to accidental overdoses; this number increased to 39% between 2017 and 2018 and is forecasted to rise [Philadelphia Maternal Mortality Review Committee (MMRC), 2020]. In Philadelphia, approximately 74% of fatal accidental overdoses occurred postpartum, with 66% occurring more than six weeks postpartum (Philadelphia MMRC, 2020). Rates of neonatal abstinence syndrome (NAS) in Philadelphia have also risen. In 2019, the rate of NAS was 12.8 per 1,000 live births, nearly double the rate reported in 2010 (Health of the City Report, 2021).

During the prenatal period, pregnant people with SUD are less likely to access prenatal health care services (Henkhaus et al., 2022; Kotelchuck et al., 2017), despite being more likely to have preexisting health conditions and health disadvantages than pregnant people without SUD (Kotelchuck et al., 2017). The year after delivery is an especially vulnerable period for people with a current or past SUD history because overdose events increase in the postpartum period (Schiff et al., 2018). According to the 2020 Philadelphia MMRC, most pregnancy-associated deaths occurred six weeks postpartum. Schiff et al. (2018) hypothesize several explanations for this situation. Among them is that less support is offered postpartum because most resources, including housing, health insurance,^1^ and medical care, are prioritized for the prenatal period. The Philadelphia MMRC recommends directing more resources for the postpartum period.

In response to the growing impact of the opioid epidemic and the unique needs of birthing people with SUD, the Philadelphia Department of Public Health developed the Doula Support Program (DSP), which offers support for up to one-year postpartum.

### The Role of Doulas in Supporting Pregnant People with SUD

A doula is a trained professional who provides nonclinical services, including emotional, physical, and informational support during labor, delivery, and the postpartum period and who also promotes self-advocacy (Thomas et al., 2017). Doula care is associated with lower maternal stress, improvements in birth outcomes, including lower rates of cesarean section and increased satisfaction with the birth experience (Kozhimannil et al., 2014). Doula care is a promising approach to supporting birthing people with SUD, because many birthing people with SUD benefit from accurate information on how substance use can affect perinatal outcomes and pain management, as well as skills to improve communication with their provider (Alexander et al., 2021; Martin et al., 2022; O’Rourke-Suchoff, 2020; Van Boekel et al., 2013). Gannon et al. (2022) examined the experiences of people with SUD who received doula care during the perinatal period in Philadelphia, including DSP clients. Their study revealed doula engagement to be linked with increased perceptions of emotional support, health literacy, recovery, and advocacy.

### The Philadelphia Department of Public Health Doula Support Program

The DSP is a program of the Philadelphia Department of Public Health’s Division of Maternal Child and Family Health and is funded by the federal Title V Maternal and Child Health Block Grant Program.^2^

During the formation period of the DSP, designated staff at the division of Maternal, Child and Family Health consulted with the local community-based doula program, Maternity Care Coalition, and convened focus groups with the coalition’s network of doulas. They also held discussions with the Brooklyn Healthy Start doula program, By My Side Birth Support Program, which also informed the DSP development. The Brooklyn-based program combines aspects of private doula practice and community-based programs to offer an expanded scope of services including screening for depression, food insecurity, intimate partner violence, and medical risk factors (Thomas et al., 2017).

**Fig. 1:**
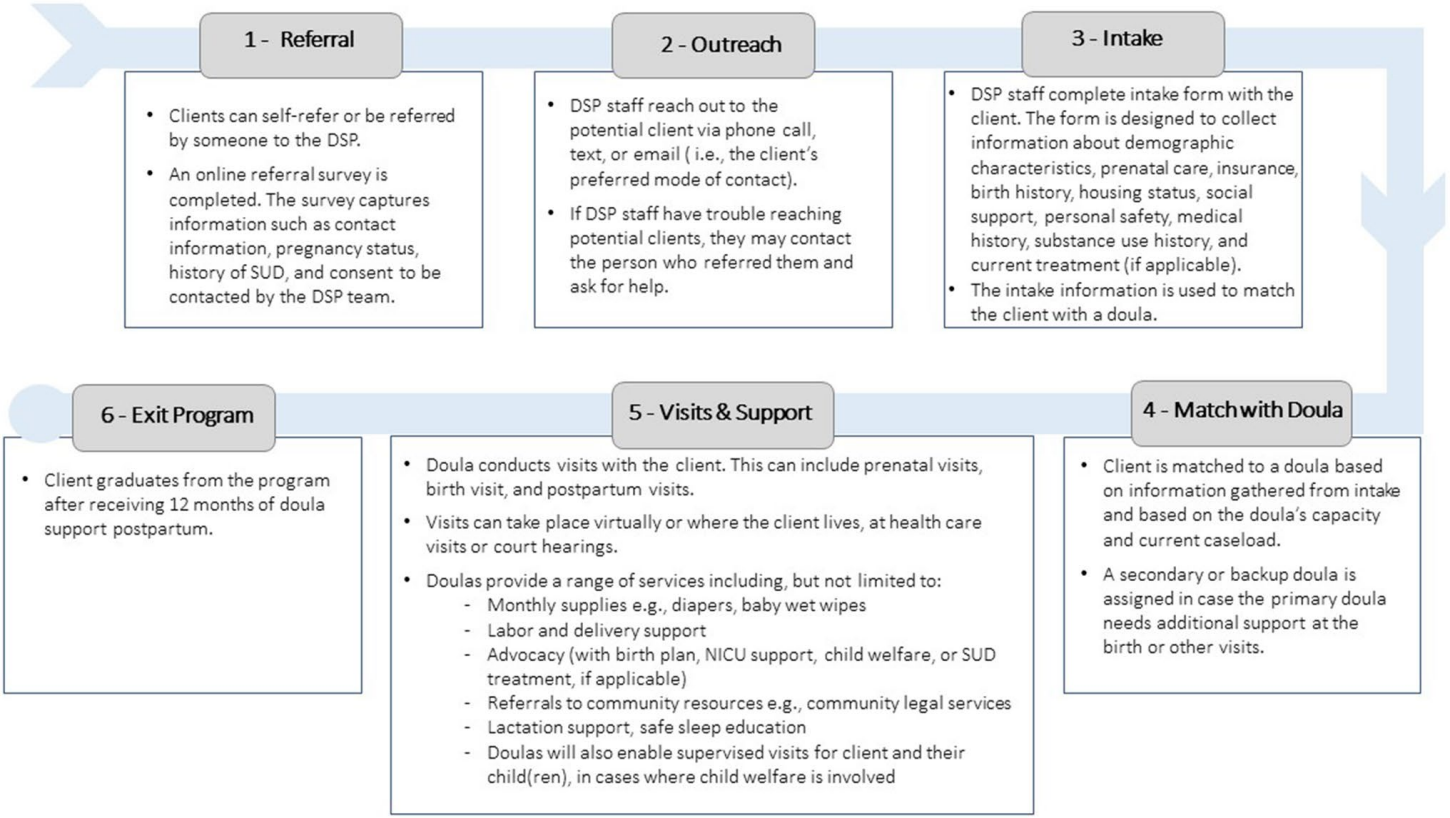
Five steps in engaging birthing people in doula care and services provided

Based on insights from the Philadelphia and Brooklyn doula programs, the Division of Maternal, Child and Family Health developed the DSP using a community-based doula model, which is an expanded approach to traditional doula care involving more frequent visits between the client and doula and a wider array of services (Bey et al., 2019). The DSP offers free, nonjudgmental support throughout pregnancy, birth, and up to 12 months postpartum. Figure 1 outlines the steps involved in engaging birthing people in the DSP and lists the support doulas offer beyond the scope of traditional doula care.

Although the DSP was designed with a focus on pregnant people with a past or current SUD, the program is open to all pregnant people in Philadelphia who desire doula care. There are no program requirements and active substance use does not disqualify a person from the program. Substance use should be viewed as a coping mechanism for trauma, rather than a moral failing, and recovery is acknowledged as a nonlinear journey, especially for parents of newborns (National Harm Reduction Coalition, 2020). The DSP aims to minimize stigma and judgment by treating clients with dignity and respect, to support them in meeting their treatment and parenting goals through nonpunitive approaches.

## Methods

A group interview with DSP founding members was convened to discuss challenges and successes of the program’s implementation. A semi-structured format was chosen to allow each interviewee to respond based on the importance and relevance of each topic (Green & Thorogood, 2009). The interview guide (Supplementary Material) was designed to capture the program’s trajectory from conception to implementation. The interview was audio recorded and transcribed.

A priori codebook including descriptive codes was developed and refined to incorporate emerging themes. Documented early discussions and memos captured during the formation and implementation periods of the program supplemented the group interview findings.

The Philadelphia Department of Public Health Institutional Review Board approved this study.

## Assessment

Findings from the group interview centered on four broad topics: doula recruitment and retention, community partnerships to promote client referrals, doula training, and client retention.

### Recruiting and Retaining Doulas by Partnering with Local Community-Based Programs

Establishing working relationships with a network of community doulas, such as the Maternity Care Coalition, and organizations serving birthing people with SUD is critical for recruiting and retaining DSP doulas. A DSP founder made a key recommendation:

*“I think if this model is to hopefully be re-created, I would say that having this relationship with a large maternal health organization and with a robust community doula network is super crucial to actually finding the doulas and also to the formation of the program overall.”*

DSP doulas were initially recruited from the Maternity Care Coalition, the local community doula program, which supported the formation of the program. Doulas have since been recruited through word-of-mouth, job postings, networking, and outreach events although most of the program doulas were recruited from the Maternity Care Coalition. To date, the DSP has successfully recruited nine doulas, only one of whom has left the program since its formation. Doula retention is important to the program’s success since it supports continuity of care and fosters positive client-doula relationships.

### Partnering with Local Organizations and Treatment Centers to Maximize Client Referrals

To date, most client referrals have come from SUD treatment programs. Other referral sources include sheltering organizations and social workers. Clients can also self-refer. Interviewees noted that establishing working relationships with organizations serving pregnant people with SUD is a priority. The program initially partnered with a maternal substance use research and treatment program for client referrals. However, DSP staff have worked to create a diverse pool of potential referral partners through networking and outreach activities. Although their promotion efforts have raised the program’s profile, referral partners are primarily treatment programs. The program acknowledges that this referral model is not appropriate for reaching and engaging pregnant people who are still using substances, as articulated by a program founder:

*“I think one of the big challenges that we still have two and a half years later is that we don’t really have a model of engaging people who are pregnant but still using drugs. Like as far as meeting them on a street level…I wanted that to be a big part of how we got people enrolled but it’s really a challenge because you just really need so much time, just to sit with someone, take them to the ER if they’re willing or ready. I just didn’t have the capacity to do that, while also getting the program up and running.”*

To address this issue, the program is focused on expanding its partnerships with shelters and creating partnerships with local harm reduction programs, emergency rooms, and providers.

### Providing Comprehensive and Ongoing Doula Training Responsive to Doulas’ and Clients’ Needs

Community health experience, familiarity with the city landscape and the local perinatal SUD system, and attendance at a minimum of 10 births are requirements for becoming a DSP doula. Doulas in this program also must have experience with people who have SUD, but not necessarily in a professional capacity. This requirement ensures that doulas have the compassion and understanding required for the role. The DSP program initially relied on the Maternity Care Coalition to develop basic trainings, but began to supplement the training with additional topics as the program grew and doulas identified challenges they encountered in the field:

*“…Fast forward, maybe a year later when we have doulas and they’re working with clients, the areas of training just emerged as necessary. I would say trainings started before [the program coordinator] joined the team, but then certainly increased after she did. With all her work with the doulas, she said there’s a lot of crises that the doulas are navigating with participants; we need crisis response training. Looking into that, and so many instances of things happening for the first time, she said, ‘we could really use some additional training about how to navigate this issue.’”*

Anecdotal evidence from program clients also informed and identified training areas. One of the group interviewees described how trainings were enriched by the doula-client relationship:

*“I would say it’s a collaborative thing in the sense that doulas will ask questions or have issues come up and maybe they’re not thinking, ‘we need training on this,’ but then [the program coordinator] and I said we do need training. For example, at [a treatment center] program, the majority of their patients that were being referred to us were on medication-assisted treatment.*

*... We learned on the job that, first of all, [methadone] is not the only medication that you can be prescribed while pregnant and postpartum...So we have the anecdotal evidence from participants sharing with their doulas how much they’re struggling on methadone postpartum, and then we have programs saying, ‘Oh no, we don’t prescribe that.’ I think our program exists in this in-between space a lot of times where we just wouldn’t learn the things that we’re learning if we weren’t experiencing them with participants.”*

The DSP coordinator identifies training opportunities, which are delivered primarily online and are either live or asynchronous teachings hosted by local, state, and national organizations. All doulas must complete these trainings. Trainings are categorized into four primary competency areas:

1. Medical literacy (e.g., understanding of medical terminology and interventions and the effects of medications such as suboxone and buprenorphine).
2. System-level training (e.g., understanding of city and hospital policies related to perinatal SUD).
3. Interpersonal skills (e.g., education on harm reduction, trauma-informed care, and crisis response).
4. Infant care (e.g., education on safe sleep and breast/chest feeding practices).

Identifying and developing the program’s trainings, most notably the medical literacy and system level trainings, has been described as a particularly challenging, but necessary, aspect of program development. When describing the initial steps involved in setting up the program, one interviewee shared:

*“You have to know the (medical) terms, interventions, the medications, not to offer advice about them, but to really just be literate in the room when things are happening. I knew that there was so much to learn about different hospital policies and there was not a roadmap for that, so that was definitely a big challenge.”*

### Addressing Retention Challenges with a Client Base that is Highly Engaged but Frequently Lost to Follow-up

Although the DSP was designed with a focus on pregnant people with a past or current SUD, the program is open to all pregnant people in Philadelphia who desire doula care. Preliminary program data indicate that clients with SUD are more likely to be lost to follow-up than clients without SUD. Individuals with SUD may be lost to follow-up for many reasons, including logistical challenges such as relocation or restricted use of their phone depending on the rules of their housing, if applicable. During the group interview, one interviewee stated that difficulties with follow-up and client retention are often mischaracterized as an individual’s inattention, rather than as a manifestation of SUD:

*“It’s often characterized as an individual’s flakiness or, you know, inconsistency. But I know from working with people living on the street and engaging in street economies and [who are] just really, in the depths of addiction, that engagement and services need to be incentivized. [People] need to be offered [something] beyond just ‘we have this service for you,’ because that’s not how the physiology of addiction and dependence on fentanyl works. It doesn’t matter how much somebody wants to participate in something, it’s just not that simple.”*

Although clients with SUD were more likely to be lost to follow-up, if clients with SUD remained in the program, they had a higher number of visits with their doula compared to clients without SUD. Even clients who resumed substance use, which typically occurred around three months postpartum, continued to engage with doula services. This demonstrates high engagement among clients with SUD.

It is also important to acknowledge that the COVID-19 pandemic made client retention challenging.

## Conclusion

There is evidence to suggest that a doula can support and advocate for pregnant people with SUD and improve maternal health. The Philadelphia Department of Public Health DSP offers an expanded model of doula care for up to one-year postpartum. It is too early to determine the long-term impact of the DSP and its effect on pregnancy-associated deaths due to accidental overdose, sustained recovery from an SUD, and maternal health. However, our initial reflections of this program show it is promising in offering support to pregnant people with SUD. DSP clients who are not lost to follow-up are highly engaged in the program and receive multiple visits within the critical six-month postpartum period. No incidents of accidental overdose have been reported among the client population, and long-term engagement with the program has enabled doulas to support a client’s recovery when incidents of resumed substance use have occurred.

Future research directions include analyzing program data to assess whether easing COVID-19 restrictions and resuming in-person visits has resulted in improved client retention. To understand and improve client engagement with the program, the perspectives of the DSP clients will continue to be sought. Future steps to assess the impact of the DSP include continued research on outcomes among clients with SUD and their infants. For example, authors of this paper will compare maternal and infant health outcomes between birthing people with SUD who engage in the DSP to birthing people with SUD who are not enrolled in the DSP.

The goal of this paper has been to provide a description of the DSP and its formation, and to discuss early challenges and successes of program implementation. In doing so, we offer a model for other organizations serving pregnant people with SUD, with a particular focus on incorporating doula care.

### Supplementary Information

Below is the link to the electronic supplementary material.

Supplementary file1 (DOCX 16 KB)

## Data Availability

Not applicable.

## Footnotes

1 A direct recommendation in the Philadelphia MMRC 2020 report was to extend Medicaid postpartum coverage. The Pennsylvania 2021 MMRC report also recommended extending Medicaid postpartum care. Effective April 1, 2022, Pennsylvania extended Medicaid postpartum coverage to one year postpartum.

2 The Title V Maternal and Child Health Block Grant Program is a partnership between the federal government and states. The goal of the program is to support the health and well-being of all mothers, children, and families (Health Resources and Services Administration, 2022).
