# Correction: Philadelphia Department of Health Doula Support Program: Early Successes and Challenges of a Program Serving Birthing People Affected by Substance Use Disorder

**DOI:** 10.1007/s10995-023-03855-6

**Published:** 2023-11-24

**Authors:** Nadia Haerizadeh-Yazdi, My-Phuong Huynh, Arielle Narva, Amirah Grasty, MaryNissi Lemon, Nick Claxton, Kelly Gillespie, Stacey Kallem

**Affiliations:** Philadelphia Department of Public Health, Division of Maternal, Child and Family Health, 1101 Market Street, Philadelphia, 19107 United States

**Correction to: Maternal and Child Health Journal**

10.1007/s10995-023-03803-4

The original version of this article unfortunately contained error in article first page.

The article is published with incorrect Abstract, keywords, Significance statement and corresponding author’s e-mail address. The correct version is given below.

## Abstract

### Purpose

Maternal substance use and deaths due to overdoses are increasing nationwide. Evidence suggests that the rate of resumed substance use, and fatal and non-fatal overdose is greatest in the first year after birth, particularly around six months postpartum, compared to other parts of the perinatal period. Doula care has been linked to improvements in perinatal health and outcomes.

### Description

In response to the opioid epidemic, the Philadelphia Department of Public Health developed and implemented the Doula Support Program (DSP), with a focus on one year of postpartum care for birthing people with a substance use disorder (SUD). In this paper, we describe the program and its formation and report on the early challenges and successes of the program implementation, based on information we received from program founders and managers in a group interview.

### Assessment

Early successes of the program include partnering with local community-based programs to recruit and retain doulas, supplementing traditional doula education with perinatal SUD-specific trainings, and maximizing client referrals by collaborating with local organizations and treatment centers that serve birthing people with SUD. Client retention, however, has proven to be challenging, especially during the COVID-19 pandemic.

### Conclusion

The DSP continues to grow, and lessons learned will facilitate program improvements. The goal of this paper is to outline the development and launch of the DSP and to act as a model for other state and local health departments interested in providing doula care for birthing people with SUD.

## Significance

Maternal substance use and fatal overdoses are increasing in Philadelphia, Pennsylvania. In response to this crisis, the Philadelphia Department of Public Health’s Division of Maternal, Child and Family Health developed the Doula Support Program (DSP), offering doula care to pregnant people with a current or past SUD history up to one-year postpartum. To our knowledge, this is the only doula program in Pennsylvania offering one-year postpartum support to birthing people affected by SUD.

### Keywords

Doula care, substance use disorder, perinatal, postpartum, local health department

The correct e-mail address of Corresponding Author Nadia Haerizadeh-Yazdi is nadia.h-yazdi@phila.gov

The original article has been corrected.

