# Supplementary material for: Philadelphia Department of Health Doula Support Program: Early Successes and Challenges of a Program Serving Birthing People Affected by Substance Use Disorder
Source: Matern Child Health J. 2023 Nov 9;27(Suppl 1):52–7. doi: 10.1007/s10995-023-03803-4 (PMC10692009; doi:10.1007/s10995-023-03803-4)
Supplement: Supplementary file 1 — Supplementary file1 (DOCX 16 KB) [file 10995_2023_3803_MOESM1_ESM.docx]

**Supplementary Material**

**Interview Guide for Program Managers and Founders of the Doula careDoula care Program**

| **Introduction** |
| --- |
| 1) Starting at the beginning, can you tell me a bit about what led to the creation of this program?   - When did the program launch? - What led to its inception and focus on women with SUD/OUD? - Tell me more about the one-year postpartum period focus. - What was going on in the city at the time? - How did you identify this unmet need? |
| 2) What is your background, and what did you bring to this program? |
| 3) What were some of your expectations about starting a program like this?   - What was your vision? - What was your goal in creating this program? - Did you expect any challenges? |
| **Planning and Launching the Program** |
| 4) What were some of the steps/processes that went into creating this program?   - Did you model it based on another program? - How did you identify the areas of support which the program has to offer (e.g., breastfeeding, safe sleep, and family engagement)? - Do/did you work closely with any other organizations as part of organizing and planning? |
| 5) What trainings were developed for doulas to prepare them to be a part of the program?   - What informed this training (e.g., the trainings on trauma awareness, motivational interviewing, social determinants of health, adverse childhood experiences)? |
| 6) Did you face any challenges when it came to recruiting doulas for the program?   - What were the criteria you looked for when recruiting doulas? |
| **Program Launch** |
| 7) What were some challenges that came up while implementing this program?   - Can you recall an incident? How were these challenges addressed? |
| 8) What were some successes in implementing this program?   - How do you measure this success? |
| 9) What were some supports in place for doulas working in this program? |
| **At Present** |
| 10) What are lessons learned you would share with others wanting to implement a doula program for SUD/OUD populations? |
| 11) What suggestions do you have for others working with this population? |
| 12) What are future steps or plans for this program? |
